# Compound Heterozygous p.(R124C) (Classic Lattice Corneal Dystrophy) and p.(R124H) (Granular Corneal Dystrophy Type 2) in *TGFBI*: Phenotype, Genotype, and Treatment

**DOI:** 10.3390/genes16010076

**Published:** 2025-01-11

**Authors:** Ji Sang Min, Tae-im Kim, Ikhyun Jun, R. Doyle Stulting, Changrae Rho, Sang Beom Han, Heeyoung Kim, Jinseok Choi, Jinu Han, Eung Kweon Kim

**Affiliations:** 1 The Cornea Dystrophy Research Institute, Yonsei University College of Medicine, 50-1 Yonsei-ro, Seodaemungu, Seoul 03722, Republic of Korea; jsmansae@yuhs.ac (J.S.M.); tikim@yuhs.ac (T.-i.K.); hadesdual@yuhs.ac (I.J.); 2 Institute of Vision Research, Department of Ophthalmology, Yonsei University College of Medicine, 50-1 Yonsei-ro, Seodaemungu, Seoul 03722, Republic of Korea; 3 Woolfson Eye Institute, Atlanta, GA 30328, USA; dstulting@icloud.com; 4 Saevit Eye Hospital, Goyang-si 10447, Republic of Korea; menard@hanmail.net (C.R.); msbhan@nate.com (S.B.H.); ophdaum@daum.net (H.K.); zenith716@hanmail.net (J.C.); 5 Institute of Vision Research, Department of Ophthalmology, Gangnam Severance Hospital, Yonsei University College of Medicine, Seoul 06273, Republic of Korea; jinuhan@yuhs.ac

**Keywords:** compound mutation of *TGFBI*, TA cloning, classic lattice corneal dystrophy (LCD), granular corneal dystrophy type 2 (GCD2)

## Abstract

(1) Background: The phenotypes of classic lattice corneal dystrophy (LCD) and granular corneal dystrophy type 2 (GCD2) that result from abnormalities in *transforming growth factor β-induced* gene (*TGFBI*) have previously been described. The phenotype of compound heterozygous classic LCD and GCD2, however, has not yet been reported. (2) Case report: A 39-year-old male (proband) presented to our clinic complaining of decreased vision bilaterally. A slit-lamp examination revealed corneal opacities consistent with classic LCD. Contrast sensitivity (CS) was decreased. A genetic analysis performed with commercially available real-time polymerase chain reaction (PCR) showed both homozygous classic LCD and homozygous GCD2. Sanger sequencing performed in our lab suggested compound heterozygosity for c.370C>T and c.371G>A variants, which was confirmed by the TA cloning of exon 4 of *TGFBI* and sequencing of clones. Phototherapeutic keratectomy (PTK) was performed on the right eye of the proband, and the CS improved. (3) Conclusions: Compound heterozygous classic LCD and GCD2 produces clinical findings like that of severe, classic LCD. PTK can improve VA and CS, delaying the need for keratoplasty.

## 1. Background

Classic lattice corneal dystrophy (classic LCD) and granular corneal dystrophy type 2 (GCD2) are phenotypes of p.(R124C) [1,2] and p.(R124H) [3] from *transforming growth factor β-induced* gene (*TGFBI*) variants, respectively, with autosomal dominant expression. The phenotype of classic LCD appears in the first to second decade of life as superficial, central fleck-like opacities with fine branching refractile filamentary lines [4]. The lines spread from the central to the peripheral cornea during the second and third decades of life. The central opacities become more pronounced with age, resulting in diffuse, subepithelial, ground-glass haze and dot-shaped opacities of the central cornea [2,5]. Heterozygous GCD2 appears initially as small granules, progressing to superficial whitish round patches that become discoid or ring-shaped lesions later in life. Heterozygous GCD2 can also show spiked, short linear, or long linear deposits and diffuse haze [6,7]. The thin lines in classic LCD are refractile and tend to cross each other, while the lines in GCD2 are thicker, whiter, less refractile, and less likely to cross [2,6].

There has been a report of a compound heterozygote of p.(R124C) (classic LCD) and p.(G470X) [8] presenting with a progressive loss of visual acuity since childhood; exhibiting dense, subepithelial opacities without accompanying typical lattice lines, and reports of compound heterozygotes of p.(R124H) (GCD2) with variants such as p.(H174D), p.(I247N), p.(Y88C), p.(R257P), or p.(R179X); and showing severe phenotypes [9,10]. The p.(G470X), p.(H174D), p.(I247N), p.(Y88C), p.(R257P), and p.(R179X) variants in the *TGFBI* gene do not induce an abnormal corneal phenotype when alone [8,9,10]. The phenotype of the compound heterozygous classic LCD and GCD2, however, has not yet been reported.

We report a case of compound heterozygous classic LCD and GCD2, the results of genetic diagnostic procedures, and the outcome of phototherapeutic keratectomy (PTK).

## 2. Case Presentation

### 2.1. Clinical Analysis

A 39-year-old Korean male (proband) presented with decreased corrected distance visual acuity (CDVA) of 20/200 (OD) and 20/150 (OS). The patient had previously experienced painful recurrent erosions but had no history of trauma or ocular surgery.

**Figure 1:**
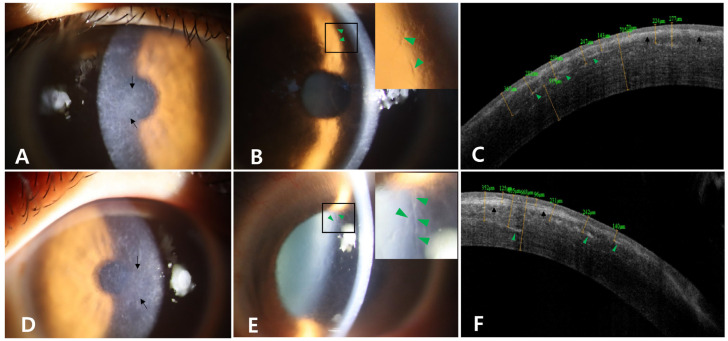
Slit-lamp photographs and Fourier-domain anterior segment optical coherence tomographs (FD-OCT) of the proband. Slit-lamp photographs of the right eye (**A**,**B**) and left eye (**D**,**E**) are also shown. Diffuse grayish-white opacities in the subepithelial and superficial stroma ((**A**,**D**); black arrows) and distinct refractile lattice lines spreading to the periphery ((**B**,**E**); magnified views are shown at the upper right) were observed in both corneas of the proband. FD-OCT images of the right eye (**C**) and left eye (**F**) are shown. Diffuse grayish-white opacities in the subepithelial and superficial stromal area (black arrowheads) and many distinct refractile lattice lines spreading to the periphery (green arrowheads) were observed.

**Figure 2:**
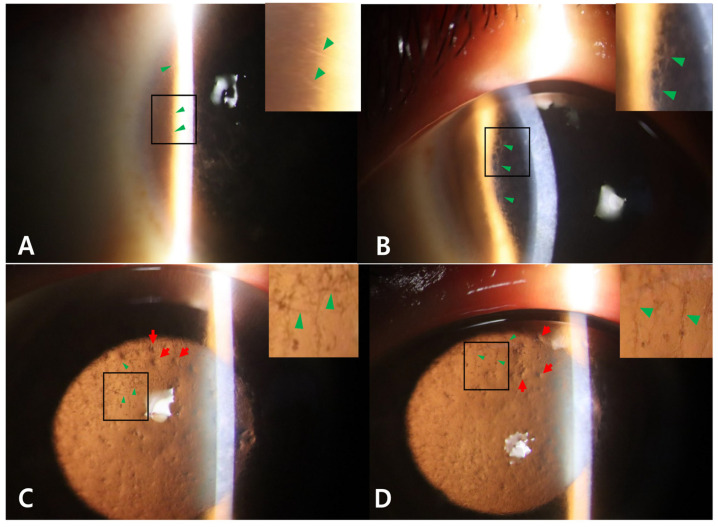
Slit-lamp photographs of the right eye (**A**) and left eye (**B**) of the proband are shown. Many distinct refractile lattice lines spreading to the periphery near to the limbus (green arrowheads) were observed (**A**). Dense, thick lattice lines are seen in the mid-peripheral cornea (**B**). Slit-lamp photographs using retro-illumination of the right eye (**C**) and left eye (**D**) show 35 lines in the temporal half OD and 33 in the nasal half OS. Many dot-shaped opacities (40 OD and 23 OS) under retro-illumination (red arrows) and distinct refractile lattice lines spreading to the periphery (green arrowheads) were observed in both corneas of the proband (magnified views are shown at the right upper corners).

Both of the proband’s corneas exhibited an irregular surface and diffuse grayish-white deposits in the subepithelial and superficial stroma along with distinct refractile lattice lines spreading to the periphery (Figure 1A,B,D,E). Fourier-domain anterior segment optical coherence tomography (FD-OCT) (RTVue-100; Optovue Inc., Fremont, CA, USA) confirmed that the proband had thick central superficial stromal opacities (Figure 1C,F). The lattice lines of the proband were more numerous in the mid-periphery, reaching the limbus and visible with direct and retro-illumination (Figure 2A–D). Under retro-illumination, approximately 35 lattice lines were observed in the temporal half of the proband’s right cornea, and approximately 33 lattice lines were seen in the nasal half of his left cornea (Figure 2C,D). Approximately 40 dot-shaped opacities were present in the proband’s right cornea, and approximately 23 were present in his left cornea (Figure 2C,D).

**Figure 3:**
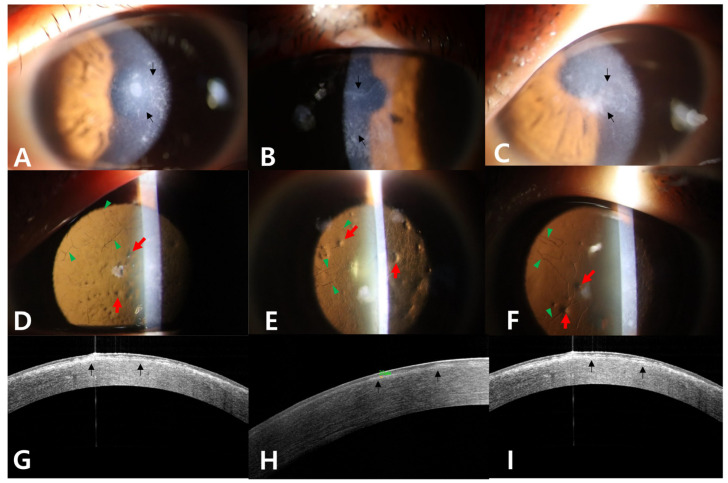
Slit-lamp photographs and Fourier-domain anterior segment optical coherence tomographic (FD-OCT) images of genetically confirmed classic LCD heterozygotes ((**A**,**D**,**G**); 32-year-old) ((**B**,**E**,**H**); 43-year-old) ((**C**,**F**,**I**); 40-year-old). Diffuse grayish-white opacities in the subepithelial and superficial stromal area (black arrows) are shown (**A**–**C**). Distinct refractile lattice lines (green arrowheads), which are less confluent (15, 12, and 14 lines in each half cornea of (**D**–**F**)) and thinner than those of the proband, spreading to the periphery are observed (**D**–**F**). The dot-shaped opacities are less numerous than those of the proband (13, 9, and 5 dots in each (**D**–**F**)) (red arrows). FD-OCT images of heterozygotes show fewer lattice lines (green arrowheads), while diffuse grayish-white opacities in the subepithelial and superficial stromal area (black arrows) were observed at various depths (**G**–**I**).

**Figure 4:**
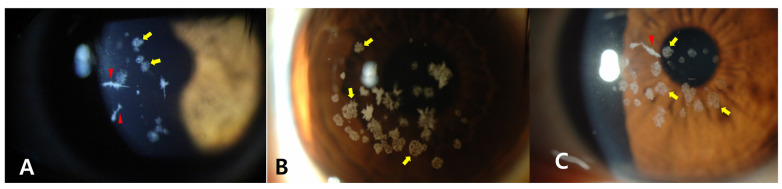
Slit-lamp photographs of other genetically confirmed GCD2 heterozygotes. Granular deposits (yellow arrows) and linear deposits (red arrowhead) were observed in the corneas of 40-year-old (**A**), 38-year-old (**B**), and 37-year-old heterozygotes (**C**).

When the findings of this case are compared to those of classic LCD heterozygotes (Figure 3) or GCD2 heterozygotes (Figure 4) at similar ages, the proband did not show the characteristics of GCD2, such as white granules, thick opaque linear opacities, and diffuse haze.

LCD recurred in the corneas of the proband’s mother following penetrating keratoplasty, which was performed 14 years ago (OD) and lamellar keratoplasty, which was performed 3 years ago. No information is available concerning the proband’s father, who is deceased.

This study was approved by the Institutional Review Board of Yonsei University College of Medicine (No. 4-2012-0209 and No. 4–2024-0794) and followed the tenets of the Declaration of Helsinki. Written informed consent was obtained from all participants.

### 2.2. Genetic Analysis

#### 2.2.1. Commercially Available Real-Time PCR

When the proband visited us initially, real-time polymerase chain reactions (PCRs) to detect *TGFBI* variations in the most common five single nucleotide polymorphisms (SNPs) (p.(R124H), p.(R124C), p.(R124L), p.(R555W), and p.(R555Q)) were requested (Avellino Labs USA). The procedures performed in Avellino Labs USA were as follows:

A DNA extraction kit (ThermoFisher, Waltham, MA, USA #4403319) was used to extract DNA from the buccal swab sample. Two sequential real-time PCR tests were performed. For the first step, a real-time PCR test was performed using probes for the detection of variant sequences for p.(R124H), p.(R124C), p.(R124L), p.(R555W), and p.(R555Q) as well as two probes for the detection of R124 and R555 normal sequences.

A signal for normal sequences only would be interpreted as normal. If there is a signal detecting any variation, second-step real-time PCRs are performed using probes for each of the 5 SNPs and the normal sequence. A homozygote is defined by detection of a single nucleotide variation only, and a heterozygote is defined by detection of both abnormal and normal sequences.

The real-time PCR report from the Avellino Labs USA for the proband showed a homozygote of classic LCD and a homozygote of GCD2. This is from the automatic report system of Avellino Labs USA identifying it as a homozygote of classic LCD when a classic LCD probe sequence only was detected, but there was no normal sequence for R124. When another real-time PCR was performed with a GCD2 probe and a normal sequence of R124, only the GCD2 sequence was detected, which identified him as a homozygote of GCD2. The commercial genetic testing report was, therefore, incorrect because an individual cannot have two different homozygous variants of the same allele.

#### 2.2.2. Sanger Sequencing: Genomic DNA Preparation and Variation Analysis

**Figure 5:**
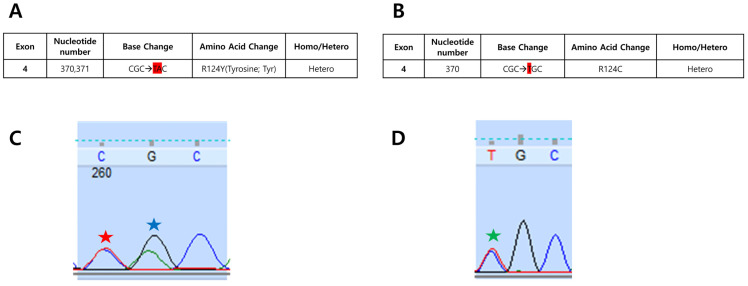
Genetic analysis results of *TGFBI* in the proband (**A**,**C**) and proband’s mother (**B**,**D**). The nucleotides changed from normal sequence are shown in red highlight (**A**,**B**). The automatic reader reported a change of arginine to tyrosine in the proband (**A**) and a change of arginine to cysteine in the proband’s mother (**B**). Partial nucleotide sequences of exon 4 of the *TGFBI* gene of the proband shows both C and T curves at 370 nucleotide (red star) and both C and A curves at 371 nucleotide (blue star) (**C**). Partial nucleotide sequences of exon 4 of the *TGFBI* gene of the proband’s mother shows both C and T curves at 370 nucleotide (green star) (**D**).

To resolve this conflict, Sanger sequencing of the whole cDNA of *TGFBI* (NM_000358.3) from the peripheral blood was performed in the Corneal Dystrophy Research Institute as described previously [11]. Sanger sequencing of the proband showed two heterozygous changes in exon 4, c.370C>T and c.371G>A variants in *TGFBI* (Figure 5A,C). Sanger sequencing was performed two additional times to rule out the possibility of errors in the procedures, and identical results were obtained. Sanger sequencing of the proband’s mother revealed a heterozygous c.370C>T:p.(R124C) variant (classic LCD heterozygote), suggesting that the two variants were on different alleles, compound heterozygous, in the proband. (Figure 5B,D). Since the proband’s father had passed away, no biologic specimen was available from him for sequencing. The c.370C>T variant has been listed with a minor allele frequency (MAF) of 3/1613794 in gnomAD v4.1, and this variant is absent in both gnomAD v4.1 East Asian and Korean Reference Genome Database (KRGDB) [12]. This variant is predicted to be deleterious in silico (AlphaMissense: 0.6908, CADD: 28.9, SIFT: 0.00, PolyPhen2: 0.958) [12]. The MAF of c.371G>A variant is 36/1613854 in gnomAD v4.1, 16/44870 in gnomAD v4.1 East Asian, and 11/10508 in KRGDB. The c.371G>A variant is predicted to be deleterious in silico except for AlphaMissense (AlphaMissense: 0.166, CADD: 26.6, SIFT: 0.01, PolyPhen2: 0.958) [13]. These two variants are located in functional FAS1 and TGF-beta/periostin domains [14,15].

The finding of a CGC substitution for TAC at the R124 site of the proband would be possible if the mother’s CGC → TGC (cystine; classic LCD) and the father’s CGC → CAC (histidine; GCD2) were transmitted as trans-compound traits. It remains also possible that the proband has a de novo mutation of TAC. We, therefore, devised a method to confirm the proband’s TAC origin.

#### 2.2.3. TA Cloning, Colony PCR, and Direct Sequencing

To investigate whether the two *TGFBI* variants are on the alleles of different chromosomes or on one allele in a single chromosome in the proband, allele-specific cloning and sequencing testing, including TA cloning, colony PCR, and direct sequencing, was requested from a commercial lab (Cosmogenetech, Seoul, Republic of Korea). Genomic fragments of exon 4 for TA cloning were obtained with the PCR condition (an initial denaturation step at 95 °C for 5 min; 35 cycles at 95 °C for 1 min, 58 °C for 30 s, and 72 °C for 30 s; and a final extension step at 72 °C for 5 min using primer (Forward, 5′-GTTCACGTAGACAGGCATTTGA-3′, Reverse, 5′-GCCTTTTCTAAGGGGTTAAGGA-3′)).

TA cloning, colony PCR, and direct sequencing were performed with the use of pMD20 T vector (2763 bp; Takara bio, Shiga, Japan) and with the Universal primer (pK19-F, 5′-CTTCCGGCTCGTATGTTGTG-3′; pK19-R, 5′-CCTCTTCGCTATTACGCCAG-3′). The detection of the c.370C>T:p.(R124C) variant 10 times and the detection of the c.371G>A:p.(R124H) variant 14 times in 24 tests confirmed that these two variants were present in trans positions.

### 2.3. Phototherapeutic Keratectomy (PTK)

**Figure 6:**
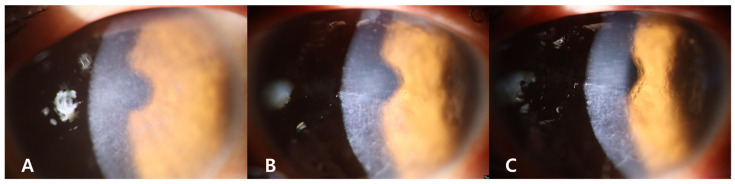
Slit-lamp photographs of the proband’s right eye before PTK (**A**) and after PTK of 30 μm and additional 10 μm ablations (**B**,**C**) performed sequentially on the same day. Since the surgeon could not determine the depth of opacities precisely before PTK, an additional 10 μm ablation was performed with a slit-lamp examination after the 30 µm PTK to remove opacities and still preserve as much of the posterior stroma as possible for future additional PTKs.

Due to his level of visual impairment, PTK of the Bowman layer and anterior stroma of the proband’s right eye was performed following the removal of the epithelium to remove superficial corneal opacities [16]. Since the proband is relatively young, sequential PTK was performed to remove the minimal amount of stromal tissue that was required to improve visual acuity and preserve stromal tissue for repeated PTK, if needed. A 30 µm PTK was performed, and the effect was evaluated with a slit lamp in the operating room (Figure 6B). Because visually significant opacities remained, an additional 10 µm PTK was performed to remove them (Figure 6C).

**Figure 7:**
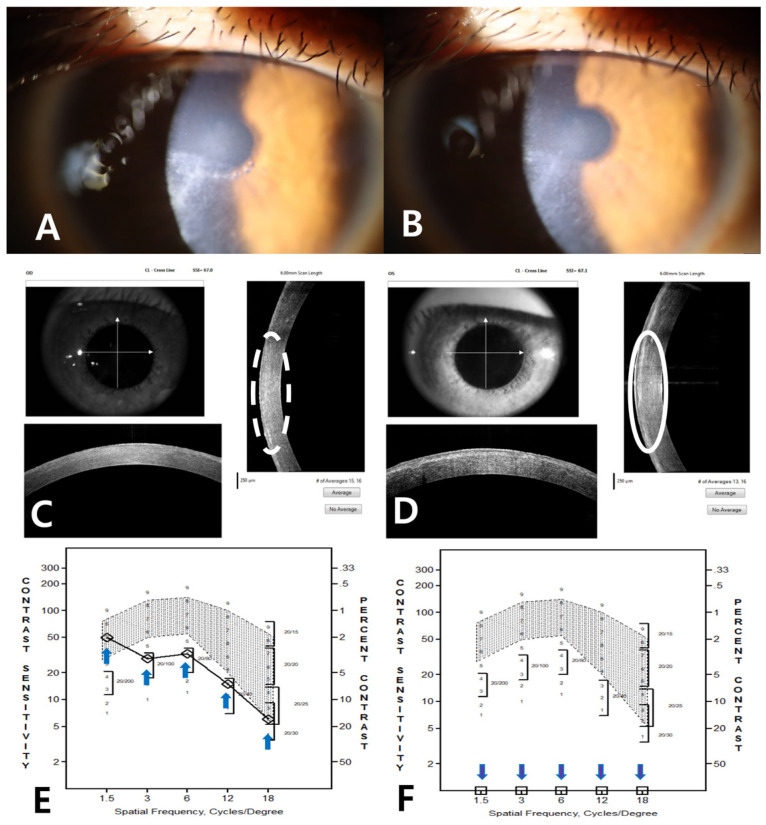
Slit-lamp photographs (OD) of the proband 1 month (**A**) and 6 months (**B**) after PTK. FD-OCT images taken 1 month after treatment show that superficial opaque deposits in the superior half were removed while some opacities remained in the inferior half of the right eye inside the white dotted oval line ((**C**), OD). Intact superficial opaque deposits remained inside the white oval line in the left eye, where PTK was not performed ((**D**), OS). The ‘# of averages’ in the FD-OCT photo refers to the number of repeated scans and automatic averaging performed by the machine as part of its noise-reduction function. The photopic contrast sensitivity testing of the right, the treated eye, of the proband 1 month after PTK showed normal values at 1.5 cycles/degree and slightly low values at other frequencies (**E**). In contrast, the left eye showed very low contrast sensitivity at all spatial frequencies (**F**).

Slit-lamp photographs and an FD-OCT image 1 month after PTK confirmed the removal of superficial opaque deposits (Figure 7A,C), but deeper linear opacities that could be seen by retro-illumination remained in the mid-periphery about 230 µm from the posterior corneal surface. His CDVA improved from 20/400 to 20/50 8 weeks after PTK and has remained stable for 6 months, with −2.75 diopters as the spherical equivalent refractive error. Corneal opacities had not recurred by 6 months following PTK.

Contrast sensitivity testing was performed on both eyes 1 month after PTK (Figure 7E,F). As can be seen, contrast sensitivity of the right eye after PTK was only slightly abnormal, while contrast sensitivity of the left, untreated eye as severely limited. (Contrast sensitivity testing was not performed prior to treatment.)

## 3. Discussion

This report describes a compound heterozygote of p.(R124C) and p.(R124H), which to our knowledge has not previously been reported. Although the available pedigree, consisting only of the proband and the proband’s mother, was limited, the mother’s p.(R124C) variant confirmed that the p.(R124H) variant came from the father, who was deceased. Although a p.(R124H) de novo variant in the proband in the allele opposite the allele containing the p.(R124C) variant is theoretically possible, its probability would be very low, even though two cases of de novo variant of p.(R124L) in *TGFBI* have been reported [17].

We found that a commercially available real-time PCR method, which detects a homozygous, heterozygous, or normal condition, can provide an incorrect result in the case of a compound heterozygote occurring in the same codon on corresponding alleles, such as this one. Since no normal sequence is detected, the report would show p.(R124C) as homozygous when p.(R124C) is examined and p.(R124H) as homozygous when p.(R124H) is examined. These interpretations are obviously incorrect since both p.(R124C) and p.(R124H) homozygous conditions cannot occur in a single individual simultaneously. We suggest that the labeling of commercially available tests for *TGFBI* variations include a warning that compound heterozygotes might be incorrectly reported as homozygotes for both variations. In these cases, TA cloning, colony PCR, and direct sequencing should be performed.

Sanger sequencing for the entire *TGFBI* sequence, repeated in triplicate to reduce the possibility of errors in the procedures (e.g., miscloning or PCR artifacts), revealed an additional problem in our case. As the sequencing data curve showed all the combined changes at once, the CGC → TGC change in the p.(R124C) variant and the CGC → CAC change in the p.(R124H) variant in the opposite allele showed a CGC → TAC heterozygous change, which can also be interpreted as tyrosine in one allele and arginine (CGC) in the opposite allele.

Due to the absence of the father’s genetic information, TA cloning, colony PCR, and direct sequencing was required to confirm the genetics of the p.(R124H) variant in this case. If the p.(R124H) variant had been detected in specimens from the father’s relatives, the correct interpretation of genetic testing would have been more obvious. Those specimens, however, could not be obtained because the proband would not allow us to contact his relatives.

Contrast sensitivity testing was not performed preoperatively, limiting our ability to determine the effect of PTK on contrast sensitivity. The contrast sensitivity of the right eye was, however, nearly normal and better than it was in the left eye, suggesting that it was improved by PTK.

The phenotype of the proband resembles that of classic LCD but features thicker central subepithelial and superficial stromal opacities. In addition, the proband’s corneas showed more pronounced lattice lines in the mid-periphery that extended to the limbus and more dot-shaped lesions that were visible by retro-illumination compared to those typical of classic LCD [18]. The phenotypic expressivity of GCD2 could not be clearly observed in this case because the manifestations of classic LCD overshadowed those that might be produced by GCD2. The reason for the dominance of manifestations of classic LCD over GCD2 is not clear and may be a result of a modifier effect or the incomplete penetration of p.(R124H).

The proband had diffuse grayish-white deposits and displayed more lattice lines extending from the central to the peripheral cornea. In cases with compound variants of p.(R124C) and p.(G470X), the cornea exhibited dense, severe subepithelial opacities without the presence of typical lattice lines [8]. Yamada et al. [9] reported a case involving a compound variant of p.(R124H) and p.(N544S), which exhibited both GCD2 and LCD phenotypes, with the GCD2 phenotype being more dominant. While only a single case of a 68-year-old male patient has been reported with the p.(N544S) variant showing late-onset atypical LCD, the p.(R124C) variant shows a severe typical phenotype with early onset. This difference in the onset and severity of clinical manifestations of p.(R124C) likely explains the discrepancy between the phenotype observed in this proband and that reported by Yamada et al. [9]. Further, in cases with compound variants of p.(R124H) and p.(H174D), severe confluent granular deposits along with dense linear deposits have been observed in the cornea [10]. The reason for a difference between the proband’s phenotype and the phenotypes of other compound heterozygotes published previously, however, is unclear, and it is necessary to evaluate the difference molecularly.

Korvatska et al. reported that amyloidogenesis in corneas with a p.(R124C) variant was accompanied by the accumulation of N-terminal kerato-epithelin fragments, with 44 kDa species as the major constituents of amyloid fibrils. Corneas in individuals with a p.(R124H) variant are characterized by predominantly non-amyloid inclusions but can show the accumulation of a 66-kDa species with the full-size 68-kDa form [4]. Courtney et al. reported that the degradation patterns are different in granular corneal dystrophy type 1 (GCD1) and classic LCD (LCD1), as 3 unique proteins were identified in GCD1 aggregates, while 18 and 24 unique proteins were isolated from stromal and Bowman’s amyloid deposits in LCD1. They also showed the presence of the HtrA1 protease in LCD1-amyloid aggregates [5]. These data show that the degradation pathways of transforming growth factor β-induced protein (TGFBIp) are different for GCD1, GCD2, and classic LCD.

We cannot determine the precise molecular mechanism by which severe classic LCD findings, but not GCD2 findings, were seen in this single case. Han et al. reported that TGFBIp expression by different-donor-derived cultured corneal fibroblasts were different from one another [2], while Choi et al. showed two-fold differences in the expression of 555 genes between wild-type and homozygous GCD2 primary cultured corneal fibroblasts [19]. Although the single case presented in this study may not represent the entire population with the same mutations, we hypothesize that the degradation of TGFBIp in this case is mainly along the classic LCD pathway so that the phenotype of classic LCD was expressed in this compound p.(R124C) and p.(R124H) variants. Further studies to determine the molecular mechanism of the compound variant of p.(R124C) and p.(R124H) are necessary in the future.

The proband’s visual acuity improved after a 40 (30 + 10) μm PTK. Even though the follow-up period has been short (6 months), recurrence has not been observed. We aimed to preserve as much of the posterior stroma as possible to allow for multiple PTK procedures in the future [20], if needed, and to delay keratoplasty. The long-term observation of the effect of PTK on contrast sensitivity will be necessary.

In summary, the p.(R124C) variant combined with the p.(R124H) variant in this case results in a severe form of the classic LCD phenotype. To our knowledge, this is the first report of clinical findings in a patient with both classic LCD and a GCD2 variant in a compound heterozygous configuration. We hope this report will provide valuable insight into the phenotype–genotype correlation of *TGFBI*-related corneal dystrophies.

## Data Availability

All data analyzed during the current study are available from the corresponding author (E.K.K.) on reasonable request.

## References

[1] Klintworth G.K. (2009). Corneal dystrophies. Orphanet J. Rare Dis..

[2] Han K.E., Choi S.I., Kim T.I., Maeng Y.S., Stulting R.D., Ji Y.W., Kim E.K. (2016). Pathogenesis and treatments of TGFBI corneal dystrophies. Prog. Retin. Eye Res..

[3] Kheir V., Cortés-González V., Zenteno J.C., Schorderet D.F. (2019). Mutation update: TGFBI pathogenic and likely pathogenic variants in corneal dystrophies. Hum. Mutat..

[4] Kannabiran C., Klintworth G.K. (2006). TGFBI gene mutations in corneal dystrophies. Hum. Mutat..

[5] Milovanova E., Gomon S., Rocha G. (2024). Classic lattice corneal dystrophy: A brief review and summary of treatment modalities. Graefes Arch. Clin. Exp. Ophthalmol..

[6] Nielsen N.S., Poulsen E.T., Lukassen M.V., Chao Shern C., Mogensen E.H., Weberskov C.E., DeDionisio L., Schauser L., Moore T.C.B., Otzen D.E. (2020). Biochemical mechanisms of aggregation in TGFBI-linked corneal dystrophies. Prog. Retin. Eye Res..

[7] Holland E.J., Daya S.M., Stone E.M., Folberg R., Dobler A.A., Cameron J.D., Doughman D.J. (1992). Avellino corneal dystrophy. Clinical manifestations and natural history. Ophthalmology.

[8] Sakimoto T., Kanno H., Shoji J., Kashima Y., Nakagawa S., Miwa S., Sawa M. (2003). A novel nonsense mutation with a compound heterozygous mutation in TGFBI gene in lattice corneal dystrophy type I. Jpn. J. Ophthalmol..

[9] Yamada N., Kawamoto K., Morishige N., Chikama T., Nishida T., Nishioka M., Okayama N., Hinoda Y. (2009). Double mutation (R124H, N544S) of TGFBI in two sisters with combined expression of Avellino and lattice corneal dystrophies. Mol. Vis..

[10] Jun I., Ji Y.W., Choi S.I., Lee B.R., Min J.S., Kim E.K. (2021). Compound heterozygous mutations in TGFBI cause a severe phenotype of granular corneal dystrophy type 2. Sci. Rep..

[11] Munier F.L., Korvatska E., Djemaï A., Le Paslier D., Zografos L., Pescia G., Schorderet D.F. (1997). Kerato-epithelin mutations in four 5q31-linked corneal dystrophies. Nat. Genet..

[12] Lee J., Lee J., Jeon S., Lee J., Jang I., Yang J.O., Park S., Lee B., Choi J., Choi B.O. (2022). A database of 5305 healthy Korean individuals reveals genetic and clinical implications for an East Asian population. Exp. Mol. Med..

[13] Schubach M., Maass T., Nazaretyan L., Röner S., Kircher M. (2024). CADD v1.7: Using protein language models, regulatory CNNs and other nucleotide-level scores to improve genome-wide variant predictions. Nucleic Acids Res..

[14] Kim J.E., Park R.W., Choi J.Y., Bae Y.C., Kim K.S., Joo C.K., Kim I.S. (2002). Molecular properties of wild-type and mutant betaIG-H3 proteins. Investig. Ophthalmol. Vis. Sci..

[15] Basaiawmoit R.V., Oliveira C.L., Runager K., Sørensen C.S., Behrens M.A., Jonsson B.H., Kristensen T., Klintworth G.K., Enghild J.J., Pedersen J.S. (2011). SAXS models of TGFBIp reveal a trimeric structure and show that the overall shape is not affected by the Arg124His mutation. J. Mol. Biol..

[16] Cennamo G., Rosa N., Rosenwasser G.O., Sebastiani A. (1994). Phototherapeutic keratectomy in the treatment of Avellino dystrophy. Ophthalmologica.

[17] Tanhehco T.Y., Eifrig D.E., Schwab I.R., Rapuano C.J., Klintworth G.K. (2006). Two cases of Reis-Bücklers corneal dystrophy (granular corneal dystrophy type III) caused by spontaneous mutations in the TGFBI gene. Arch. Ophthalmol..

[18] Lakshminarayanan R., Chaurasia S.S., Anandalakshmi V., Chai S.M., Murugan E., Vithana E.N., Beuerman R.W., Mehta J.S. (2014). Clinical and genetic aspects of the TGFBI-associated corneal dystrophies. Ocul. Surf..

[19] Choi S.I., Yoo Y.M., Kim B.Y., Kim T.I., Cho H.J., Ahn S.Y., Lee H.K., Cho H.S., Kim E.K. (2010). Involvement of TGF-{β} receptor- and integrin-mediated signaling pathways in the pathogenesis of granular corneal dystrophy II. Investig. Ophthalmol. Vis. Sci..

[20] Ashena Z., Niestrata M., Tavassoli S. (2023). Management of Stromal Corneal Dystrophies; Review of the Literature with a Focus on Phototherapeutic Keratectomy and Keratoplasty. Vision.

